# Cerebellar Cognitive-Affective Syndrome Secondary to Epstein-Barr Virus Infection: A Case Report

**DOI:** 10.7759/cureus.49855

**Published:** 2023-12-03

**Authors:** José Carlos Medina-Rodríguez

**Affiliations:** 1 Department of Education, National Institute of Psychiatry, Mexico City, MEX; 2 Department of Neurology and Neuropsychiatry, National Institute of Psychiatry, Mexico City, MEX; 3 Postgraduate Studies Division, National Autonomous University of Mexico, Mexico City, MEX

**Keywords:** neuroinfectious diseases, neuropsychological testing, mood disorders, cognition disorders, cerebellar diseases

## Abstract

Throughout history, the cerebellum was initially perceived as potentially vestigial until the 19th century. However, subsequent research illuminated its pivotal role in coordination. Over the course of the 20th century, it became predominantly associated with motor functions. Nevertheless, in the latter half of the century, Schmahmann's pioneering research expanded the understanding of the cerebellum to encompass its involvement in cognition and emotions. In light of this evolving background, the primary objective of this paper is to present a clinical case featuring a 60-year-old male with a history of Epstein-Barr virus. This patient underwent a comprehensive neuropsychiatric assessment at a tertiary care hospital, involving thorough clinical, paraclinical, and neuroimaging examinations. The extensive medical findings strongly indicate the presence of a cognitive-affective cerebellar syndrome.

## Introduction

Background

Historically, the cerebellum was once regarded as a potentially vestigial structure within the nervous system, a prevailing perspective that persisted until the 19th century. However, this perception underwent a significant shift in light of groundbreaking research during that era. Specifically, neurophysiologists, through meticulous animal studies, demonstrated that cerebellar lesions resulted in profound impairments in coordination and balance [1].

This shift in understanding represented a departure from earlier beliefs, serving as the foundation for subsequent cerebellar research. In the 20th century, additional confirmation of these initial discoveries was offered by other investigators. Consequently, for the majority of the century, the cerebellum's role was primarily perceived within the realm of motor and vestibular coordination [2].

Nonetheless, this viewpoint was later broadened by subsequent findings. In the latter half of the 20th century, pioneering research by Schmahmann began to challenge the prevailing perspective. It became evident that cerebellar structural and functional abnormalities could extend beyond the motor and vestibular domains, impacting cognitive and emotional functions as well. This updated understanding marked a significant turning point, redirecting research towards investigating the cerebellum's roles in cognition and emotional regulation, along with associated pathologies [3].

Hence, the primary objective of this paper is to present a clinical case that comprehensively covers the cerebellum's primary and non-motor characteristics. The approach involves a detailed anatomical and functional review, using the clinical case as a foundation. Furthermore, a discussion is included that links the case's findings with neuropsychiatric correlations, grounded in the latest literature. In presenting this case, strict adherence to the CAse REport (CARE) Guidelines has been maintained. Ensuring the patient's confidentiality and upholding academic integrity have been paramount, necessitating the exclusion of all personal identification information from this report.

## Case presentation

Patient's history

A 60-year-old male, residing in a major urban area in Mexico, presents with a diverse background. He has completed 12 years of education and formerly worked as a physical education teacher before retiring in 1993. Currently, he is unmarried and is right-handed. Delving into his family medical history, his mother, who suffered from type 2 diabetes and systemic arterial hypertension, is deceased. He is the youngest of five siblings, with one sibling having passed away due to complications related to a neoplasm in the nervous system. Remarkably, there are no other significant non-psychiatric health issues reported in the family.

From a psychiatric standpoint, one of his siblings exhibited symptoms suggestive of an anxiety disorder but never received a formal evaluation or diagnosis. The patient, on the other hand, does not report any known family history of neuropsychiatric conditions. His own medical history includes a previous episode of Epstein-Barr virus-induced encephalitis and cerebellitis, with no other noteworthy medical antecedents reported.

In terms of psychiatric care, the patient has been under medical supervision since 1993, initially diagnosed with type 1 bipolar disorder. This condition necessitated hospitalization in the same year. His treatment plan has included a variety of medications, further detailed in his medical history. Pertaining to substance use, he has a history of alcohol and tobacco consumption that increased in 1993.

Early phase (pre 1993)

Initially, the patient, a physical education teacher, exhibited normal global functioning. However, he began to face financial stressors related to his occupation.

Significant life changes (1993)

In 1993, the patient increased his tobacco and alcohol use. This period marked a decline in his social and familial relationships. During an altercation with criminals, it is suspected that he sustained a traumatic brain injury accompanied by anterograde amnesia. There is no clear indication of any changes in his level of consciousness. Additionally, specifics concerning the mechanism of injury, any associated symptoms, or the extent of the injury's severity have not been established. He reported being inadvertently administered an unknown substance, followed by a week of fever, palpitations, and general malaise. The details of his medical treatment during this period remain unclear, leading to his family's intervention due to his deteriorating general condition.

Family intervention and escalating symptoms (1993)

Following intervention by his family, he was described as exhibiting symptoms of disorientation, irritability, reduced sleep, increased verbosity, distrust, disorganized behavior, probable complex auditory hallucinations, and aggression. The family took him to a tertiary care hospital for further medical care, as his condition did not improve with ongoing treatment for a febrile syndrome.

Hospitalization and diagnosis (1993-1994)

During a three-month hospitalization, he was diagnosed with Epstein-Barr virus infection. Post discharge, the previously described affective and psychotic symptoms appeared to have subsided, with the exception of persistent symptoms including incoordination, balance disturbances, and possible nystagmus. These issues led to frequent falls and necessitated engagement in physical rehabilitation. The exact duration and details of this rehabilitation are unknown.

Psychiatric follow-up and stability (1994-2003)

He underwent continuous psychiatric treatment in both private and public settings. He was diagnosed in these psychiatric institutions with type 1 bipolar disorder, likely based on his medical history and due to the initially referred episode of symptoms suggestive of mood elevation and psychosis. Subsequently, he remained euthymic under various treatment regimens, including lithium, antipsychotics, and antidepressants, although the specific medications and their dosages are not known. No further manic episodes were reported during this period. It is noteworthy that the previously mentioned motor and vestibular symptoms persisted.

Gradual functional decline (2003-2021)

The patient maintained a state of euthymia; however, the objective characteristics of his affective state remain ambiguous due to the retrospective nature of the case. He exhibited symptoms of irritability, distrust, and occasional verbal aggression, alongside persistent motor symptoms, which might include vestibular dysfunction, leading to functional impairment. These were characterized by generalized ataxia, dysarthria, and nystagmus. Furthermore, he increasingly relied on his family for support with daily and instrumental activities.

Deterioration (2021-2023)

Following the death of his mother in 2021, the patient showed initial signs of bereavement, which eventually subsided. However, during this period, there was a notable decline in his affective state, as observed by his family, characterized by increased irritability, distrust, and aggression. His family's accounts suggest that he may have experienced, at minimum, persecutory delusions, although the presence of other psychotic symptoms remains unclear.

Furthermore, he exhibited a decreased interest in activities, affective dysregulation, intermittent outbursts, and hoarding behaviors. Despite these symptoms persisting even under treatment with mood stabilizers, the specific medications and their dosages are unknown. It is important to note that during this period, the patient began using a wheelchair due to the ongoing presence of generalized ataxia, indicating a significant impact on his mobility and daily functioning.

Current evaluation and assessment (2023-present)

Interestingly, despite his functional decline, the patient exhibited limited self-awareness of his condition, claiming to feel reasonably well. In response to the progression of his symptoms since his mother's death, his family sought additional neuropsychiatric care. As a result, in 2023, he was evaluated as an outpatient at a psychiatric tertiary care institution.

During this initial assessment, a marked decrease in his global functionality was evident, highlighted by his near-total dependence on family for daily activities. Neurologically, while he was alert and oriented, he presented with several challenges: scanning dysarthria, generalized ataxia, dysmetria, and dysdiadochokinesia. A mild weakness in the right shoulder, likely related to a prior injury, was also noted.

His mobility was significantly compromised, necessitating the use of a wheelchair. Accompanying this were a resting tremor and partially coherent speech, mainly due to the dysarthria. His thought process appeared circumstantial and often required guidance from the interviewer, but he did not exhibit any obvious pathological ideas. Despite describing his mood as "good," his affect was observed to be restricted. He reported no perceptual disturbances.

**Figure 1:**
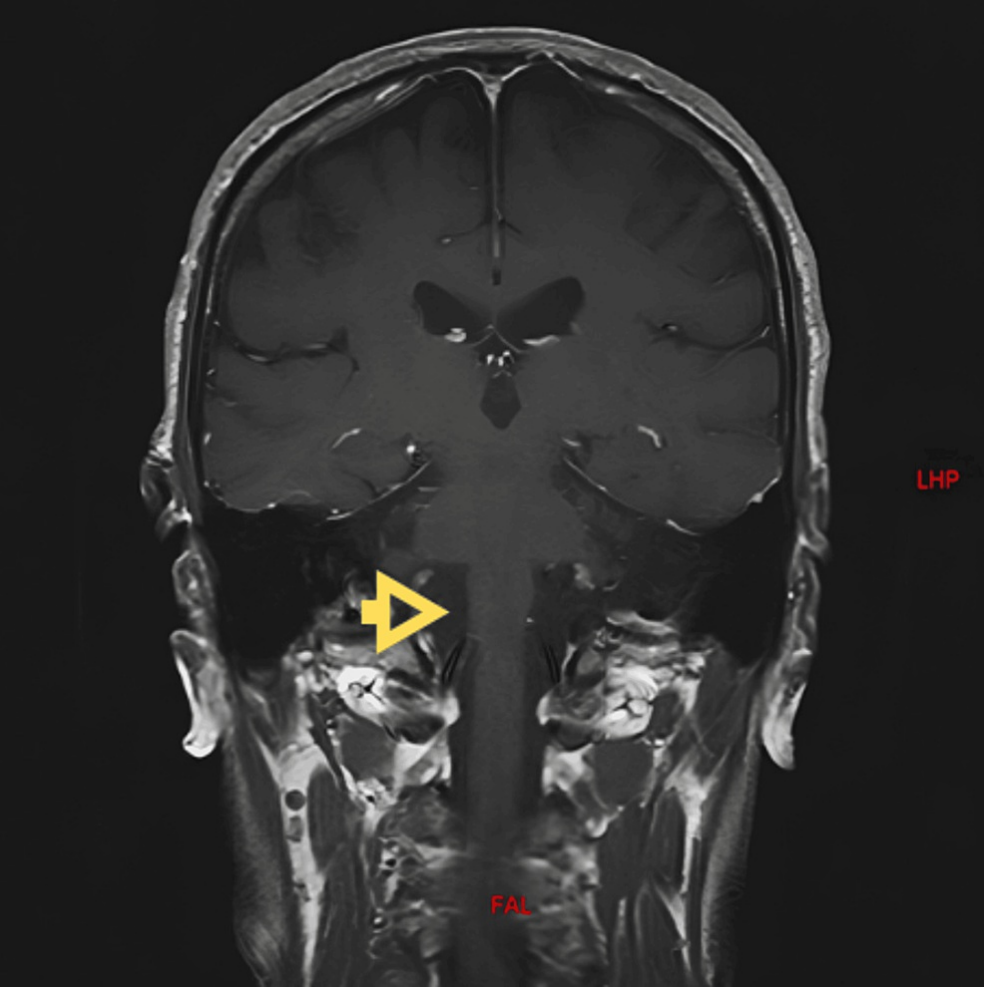
Brain MRI with gadolinium contrast enhancement in the coronal plane using a T1-weighted sequence In this image, significant bilateral cerebellar atrophy is observed, with preservation of the brainstem.

**Figure 2:**
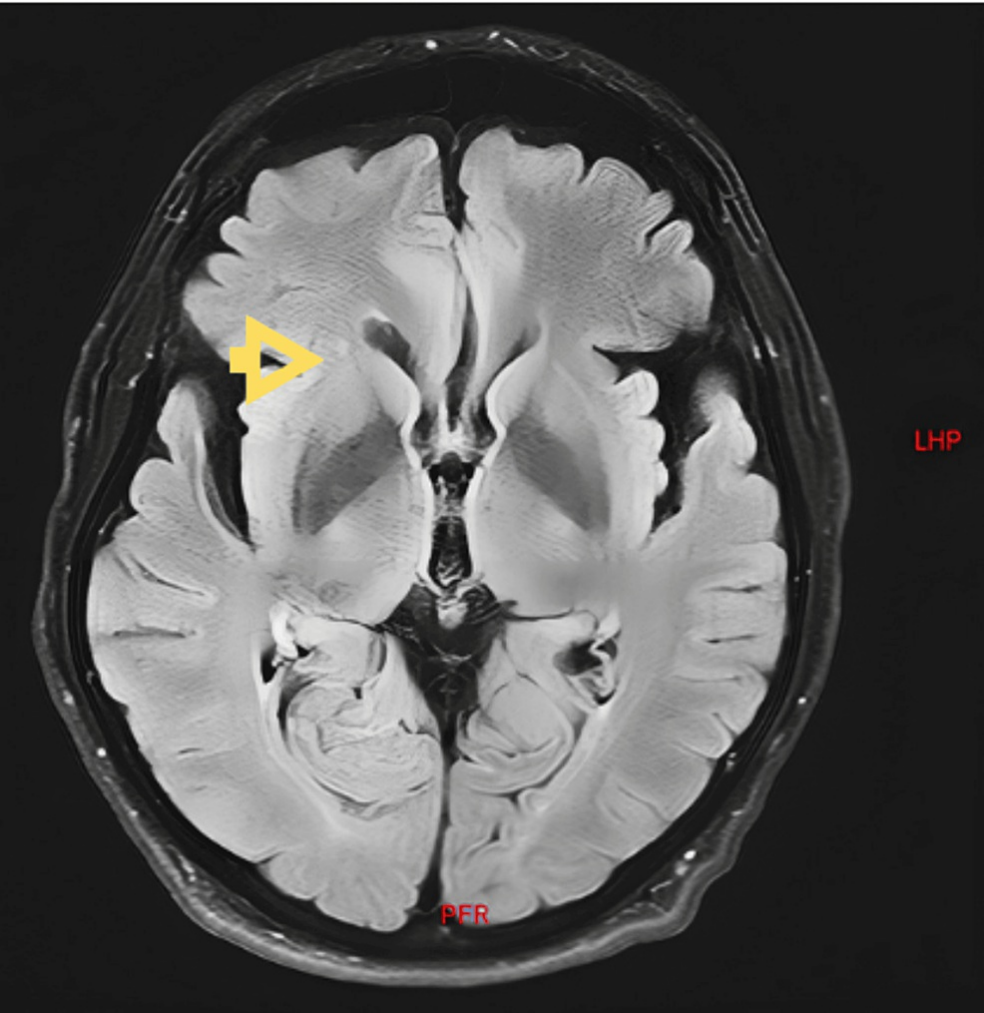
Brain MRI in T1/FLAIR sequence in axial plane In this image, a punctate subcortical right frontal hyperintensity is observed.

Laboratory tests yielded normal results, while a brain MRI showed several abnormalities, including a solitary frontal lesion and profound, diffuse cerebellar atrophy, without any evidence of gliotic or hemorrhagic lesions indicative of trauma or tumors (as depicted in Figure 1 and Figure 2).

The patient underwent a comprehensive series of neuropsychological assessments, which were known to be conducted by a specialized service with personnel trained in neuropsychological testing. However, the specific tests employed remain unknown, and only a written report of the results was available. The findings included the following:

In the domain of attention, there were significant fluctuations in his attentional resources. This was evident during tasks involving mental tracking and mental arithmetic operations. These fluctuations adversely affected his working memory, which in turn compromised his ability to perform complex daily activities. This was particularly noticeable in his inattention to topics he deemed less important.

Within the language domain, the patient's speech was characterized by spontaneity, although at times it was challenging to comprehend due to the presence of dysarthria. Overall, his verbal scale score fell within the average range. However, there was a fluctuating ability to assimilate knowledge from his environment and cultural information and apply them to solve hypothetical social dilemmas, where he exhibited certain deficits in social judgment.

His phonological verbal fluency remained intact, whereas semantic fluency showed a slight decline. He effectively named items through visual confrontation, and his vocabulary reflected a functional thought process that remained stable, without any discernible degradation in semantic fields or conceptual abilities. Regarding memory assessment, the patient demonstrated an ability to register verbal information, and this ability benefited from repetition. Nevertheless, he appeared to invest minimal effort in such paradigms, which could potentially impact his performance in information recall. Despite this, both registration and recall scores fell within the average range. Moreover, in recognition tasks, he exhibited the capacity to distinguish stored information from the rest of his lexical repository.

Considering these observed deficits in executive function, linguistic processing, spatial cognition, and affect regulation, a diagnosis of cerebellar cognitive-affective syndrome was established. The patient's care continues to be actively managed with a multidisciplinary approach, tailored to address his complex neuropsychiatric profile. Currently, he is undergoing treatment with 20 mg of fluoxetine daily for affective dysregulation, in conjunction with 4 mg of risperidone nightly, serving both as a treatment for affective symptoms and as prophylaxis against psychotic symptoms and hoarding behaviors. He has reported subjective improvement in subsequent consultations. Additionally, he is on a waiting list for more specialized physical rehabilitation to address the previously mentioned motor symptoms, and he has not engaged in the use of psychoactive substances or alcohol.

## Discussion

The medical history of this patient presents a nuanced and evolving neuropsychiatric picture. The initial years were marked by changes in lifestyle, notably an increase in alcohol and tobacco use. This period also included a traumatic brain injury with a subsequent loss of consciousness, an event that may have been instrumental in triggering a range of neuropsychiatric symptoms. Following this injury, the patient exhibited a constellation of symptoms suggestive of a manic episode, including irritability, expansiveness, reduced need for sleep, rapid speech, persecutory delusions, and behavioral disorganization, along with potential perceptual disturbances. At the same time, he experienced symptoms indicative of an acute-subacute febrile syndrome. The absence of in-depth diagnostic assessments at that time leaves the underlying etiology of these symptoms largely unconfirmed, but the clinical presentation points towards a possible encephalitic or cerebellitis syndrome, especially given the combination of altered consciousness, fever, and emergent neurological symptoms.

In the decade following 1993, type 1 bipolar disorder was diagnosed. Retrospectively, this diagnosis might have been an interpretive reach, potentially conflating the manifestations of encephalitis/cerebellitis with bipolar symptoms. Despite this, the patient achieved remission from what was perceived as manic episodes with psychotic features, indicating that the treatment regimen, though potentially misdirected, provided some symptom relief.

The year 2021 brought significant emotional disruption with the death of the patient's mother, leading to a period of bereavement that he eventually managed to navigate through. Concurrently, this phase was characterized by the development of an apathetic syndrome, which could be linked to affective or behavioral changes, alongside increased irritability, and possibly persecutory delusional thoughts. Notably, this period also saw the onset of obsessive hoarding behaviors, marking a significant shift in his psychological state. Motor symptoms consistent with cerebellar dysfunction also began to manifest, aligning with a diagnosis of motor cerebellar syndrome.

By 2023, the patient's condition had further evolved, with ongoing symptoms of motor and vestibular cerebellar dysfunction. Coupled with significant cognitive, affective, and behavioral changes, the situation led to the consideration of cerebellar cognitive-affective syndrome as a potential diagnosis.

Neuroanatomical and neuropsychiatric correlation

In terms of anatomofunctional correlation, it's worth noting that the cerebellum can be divided into three primary lobes, anterior, posterior, and flocculonodular, along with lobules I-XI, each serving distinct functions [4]. Lobules I-VI and VIII transmit sensorimotor afferents to their respective cortices via the thalamus, while lobules VI-IX are identified as the "limbic and cognitive cerebellum," exhibiting connectivity with limbic and association cortices, among others. Lastly, lobules V-VII and IX-X are integral components of the vestibular cerebellum, displaying connectivity with vestibular nuclei located in the brainstem [5].

Lesions affecting the limbic and cognitive cerebellum give rise to what is known as "Schmahmann syndrome" or cerebellar cognitive-affective syndrome. This disorder is characterized by deficits in executive function, language, affect, and various other cognitive domains. The diagnosis relies on a combination of clinical and paraclinical methods, encompassing a robust medical history review of the patient, physical examinations/neuropsychiatric assessments, psychiatric comorbidity diagnosis and treatment, physical rehabilitation, and cognitive remediation [6]. From a neuropsychological perspective, this syndrome manifests with executive dysfunction, impairments in reasoning and calculation, changes in affect, deficits in verbal and visual memory, and disturbances in language [7].

In this instance, the patient exhibited both typical motor and non-motor indications of cerebellar involvement, coupled with a medical history that hints at prior encephalitis and cerebellitis, potentially linked to the Epstein-Barr virus. This raises the possibility of post-infection cerebellar degeneration, which could entail intricate neuropathological processes akin to those observed in other degenerative conditions associated with infectious agents [8]. Given the absence of familial clustering or genetic predisposition, it is prudent to rule out hereditary cerebellar pathologies, especially in light of the seemingly preserved premorbid functionality and disease progression.

Regarding non-motor manifestations, it is noteworthy that the patient has reported experiencing symptoms aligned with existing literature, including depressive, manic, psychotic, and potentially obsessive symptoms [9]. From a cognitive perspective, there are persistent lapses in attention and challenges with immediate memory, which may indicate an executive function impairment. Although the assessment has not extensively explored language, it is reasonable to anticipate fluency issues extending beyond dysarthria, as previous studies have suggested [10]. Despite the patient's self-report and corroborating accounts from third parties pointing towards a depressive disposition, historical evidence points more towards a broader apathetic affective and behavioral syndrome, rather than an isolated depressive syndrome.

## Conclusions

This case serves as a profound illustration of the intricate and multifaceted nature of neuropsychiatric disorders. It brings to light the significant challenges faced in achieving an accurate and timely diagnosis, particularly in scenarios where neurological and psychiatric symptoms intersect and overlap. This complexity is further amplified in instances where cerebellar involvement is suspected, as it adds another layer of diagnostic ambiguity.

The case underscores the necessity of adopting a thorough and multidisciplinary approach to both the treatment and ongoing management of such conditions. This approach should ideally encompass a range of specialties, including neurology, psychiatry, neuropsychology, and rehabilitation medicine, to ensure a holistic understanding and management of the patient's symptoms.

Furthermore, it highlights the importance of considering the dynamic interplay between various neuropsychiatric symptoms and their potential cerebellar origins. This includes an appreciation of how cerebellar dysfunction can manifest not only in motor symptoms but also in cognitive and affective disturbances, thus necessitating a nuanced and tailored therapeutic strategy.

Lastly, this case exemplifies the need for continuous evaluation and adaptation of treatment plans, as neuropsychiatric conditions often present with evolving symptomatology over time. This necessitates a flexible, patient-centered approach that prioritizes the changing needs and circumstances of the individual, ensuring the best possible outcomes in terms of both symptom management and quality of life.
